# 181 Assessing the Feasibility of a 12-week Structured Exercise Programme in Improving Physical Function in Cardiovascular Disease Patients’

**DOI:** 10.1093/eurpub/ckae114.041

**Published:** 2024-09-26

**Authors:** Daire Fitzmaurice, Matthew Herring, Catherine Woods, Brian Carson

**Affiliations:** Physical Activity for Health Research Centre, Health Research Institute, University of Limerick, Ireland; Health Research Institute, University of Limerick, Ireland; Department of Physical Education and Sport Science, University of Limerick, Ireland; Physical Activity for Health Research Centre, Health Research Institute, University of Limerick, Ireland; Health Research Institute, University of Limerick, Ireland; Department of Physical Education and Sport Science, University of Limerick, Ireland; Physical Activity for Health Research Centre, Health Research Institute, University of Limerick, Ireland; Health Research Institute, University of Limerick, Ireland; Department of Physical Education and Sport Science, University of Limerick, Ireland; Physical Activity for Health Research Centre, Health Research Institute, University of Limerick, Ireland; Health Research Institute, University of Limerick, Ireland; Department of Physical Education and Sport Science, University of Limerick, Ireland

## Abstract

**Background and Purpose:**

Evidence supports the use of physical activity and exercise for the management of existing chronic disease. Exercise Referral Schemes (ERS) are one way to encourage physical activity participation in people living with a chronic disease to self-manage their own conditions and symptoms. Participants are referred by a healthcare professional to participate in a structured, supervised exercise programme. The purpose of this study was to examine the feasibility of one such scheme (ULMedX) in improving the physical function of participants with cardiovascular disease.

**Methods:**

This study utilised a pre-test post-test design (N = 29, 89.7% males; total mean age 66.17 ± 10.9 years). Participants engaged in twice weekly exercise sessions for 12 weeks focused on cardiovascular endurance and complimented by local muscular endurance training of main muscle groups. Assessments were completed at baseline and at 12-week follow up and included lower body strength (5-times sit-to-stand test), upper body strength (grip strength dynamometer), and aerobic capacity (6-minute walk test). Statistical analyses were mostly conducted using a paired-samples t-test, apart from lower body strength which used Wilcoxon-signed ranked test as data was non-normal.

**Results:**

100% of participants completed assessments at baseline with 86.21% of participants completing assessments at 12-week follow-up. The ULMedX programme had a mean attendance rate of 55.31 ± 32.22% over 12-weeks. There was a significant difference in dominant hand grip strength (p = 0.038, d = 0.37) and 5-times sit to stand scores (p = .025), but no significant difference in non-dominant grip strength (p = 0.18, d = 0.19). There was also a significant difference in the 6-minute walk test scores (p = .001, d = 0.667) with an average increase of 26.48 ± 39.72 m across all participants, which is also a clinically meaningful change.

**Conclusions:**

These findings indicate that the ULMedX exercise programme may play a significant role in improving aerobic capacity and lower limb strength for those living with cardiovascular disease. This clinically meaningful improvement could lead to an improved quality of life for these participants.

**Funding:**

Study funded by the Health and Wellbeing Division of the Health Service Executive in Community Healthcare Organisation 3.

